# G2/M checkpoint plays a vital role at the early stage of HCC by analysis of key pathways and genes

**DOI:** 10.18632/oncotarget.19351

**Published:** 2017-07-18

**Authors:** Li Yin, Cuifang Chang, Cunshuan Xu

**Affiliations:** ^1^ College of Life Science, Henan Normal University, Xinxiang 453007, Henan Province, China; ^2^ State Key Laboratory Cultivation Base for Cell Differentiation Regulation and Henan Bioengineering Key Laboratory, Henan Normal University, Xinxiang 453007, Henan Province, China; ^3^ Luohe Medical College, Luohe 462002, Henan Province, China

**Keywords:** early HCC, G2/M checkpoint, leading edge analysis, IPA, GSEA

## Abstract

The present study was designed to explore the molecular mechanism at the early stage of hepatocarcinoma (HCC) and identify the candidate genes and pathways changed significantly. We downloaded the gene expression file dataset GSE6764 from GEO, adopted the Robust Multi-array Average (RMA) algorithm to preprocess the raw file. 797 differentially expressed genes (DEGs) were screened out based on the SAM method using R language. Ingenuity Pathway Analysis (IPA) was used to perform canonical pathway analysis in order to calculate the most significantly changed pathways and predict the upstream regulators. In order to confirm the results from the DEGs which based on the individual gene level, the gene set enrichment analysis (GSEA) was done from the gene set level and the leading edge analysis was performed to find out the most appeared genes in several gene sets. The PPI network was built using GeneMANIA and the key genes were calculated using cytoHubba plugin based on cytoscape 3.4.0. We found that the Cell Cycle: G2/M DNA damage checkpoint regulation is the top-ranked pathways at the early stage of HCC by IPA. The high expression of several genes including CCNB1, CDC25B, XPO1, GMPS, KPNA2 and MELK is correlated with high risk, poor prognosis and shorter overall survival time in HCC patients by use of Kaplan-Meier Survival analysis. Taken together, our study showed that the G2/M checkpoint plays a vital role at the early HCC and the genes participate in the process may serve as biomarkers for the diagnosis and prognosis.

## INTRODUCTION

Hepatocellular carcinoma (HCC) is the fifth most common cause of cancer and responsible for a third of the cancer-related deaths worldwide. The occurrence of HCC comprises many changes such as gene mutations, chromosomal aberrations and molecular pathways which always accompanied by cell cycle dysregulation, evasion of apoptosis [1]. So far, the best therapeutic approach for the HCC patients is liver transplantation which can eliminate HCC. However, recurrence rates remain high. Methods for early HCC detection are often evaluated on specificity and sensitivity [2] and many guidelines have been established for the early liver cancer diagnosis [3].

To identify potentially useful biomarkers and targets for the early diagnosis of HCC, the molecular mechanism of the cancer has been studied intensely especially the onset of HCC [4–8]. SPRTN could decrease DNA replication stress in DNA replication and G2/M-checkpoint regulation and the mutation of SPRTN could cause early onset of hepatocellular carcinoma [9]. In order to determine candidate genes and the most significant pathways associated with the early stage of HCC, we performed the individual and gene set level analysis by use of a series of bioinformatics approaches. Especially, the differential expressed genes (DEGs) were screened out using the SAM method and the pathways enrichment was performed using Ingenuity Pathway Analysis (IPA). Furthermore, in order to avoid the drawback of individual gene analysis, GSEA was performed to verify the former result. Then, we built the PPI network from DEGs to identify the key genes using cytoHubba plugin. And then the co-expression network was built from the key genes by use of the geneMANIA plugin based on Cytoscape.

## RESULTS

### Microarray analysis and data pre-processing

**Figure 1:**
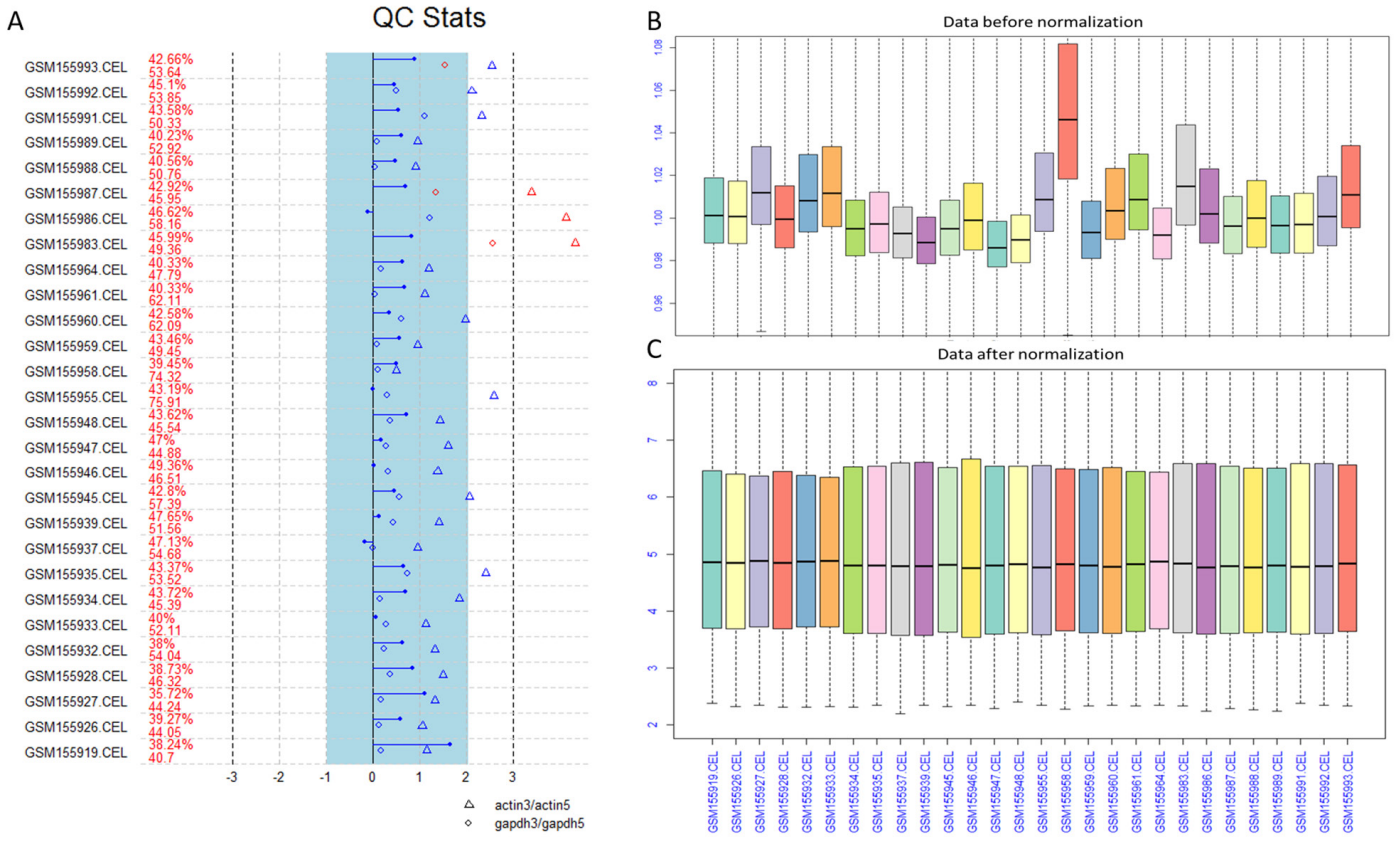
The QC plot and box plot before and after normalization **(A)** The quality control (QC) plot analysis of the raw data. **(B)** The box plot for the data before normalization. **(C)** The box plot for the data after normalization.

In order to guarantee the quality of every chip before the next analysis, we performed quality control (QC) for every raw file. The results of QC plot and box plot before and after normalization were shown in Figure 1.

### Identification of DEGs

**Figure 2:**
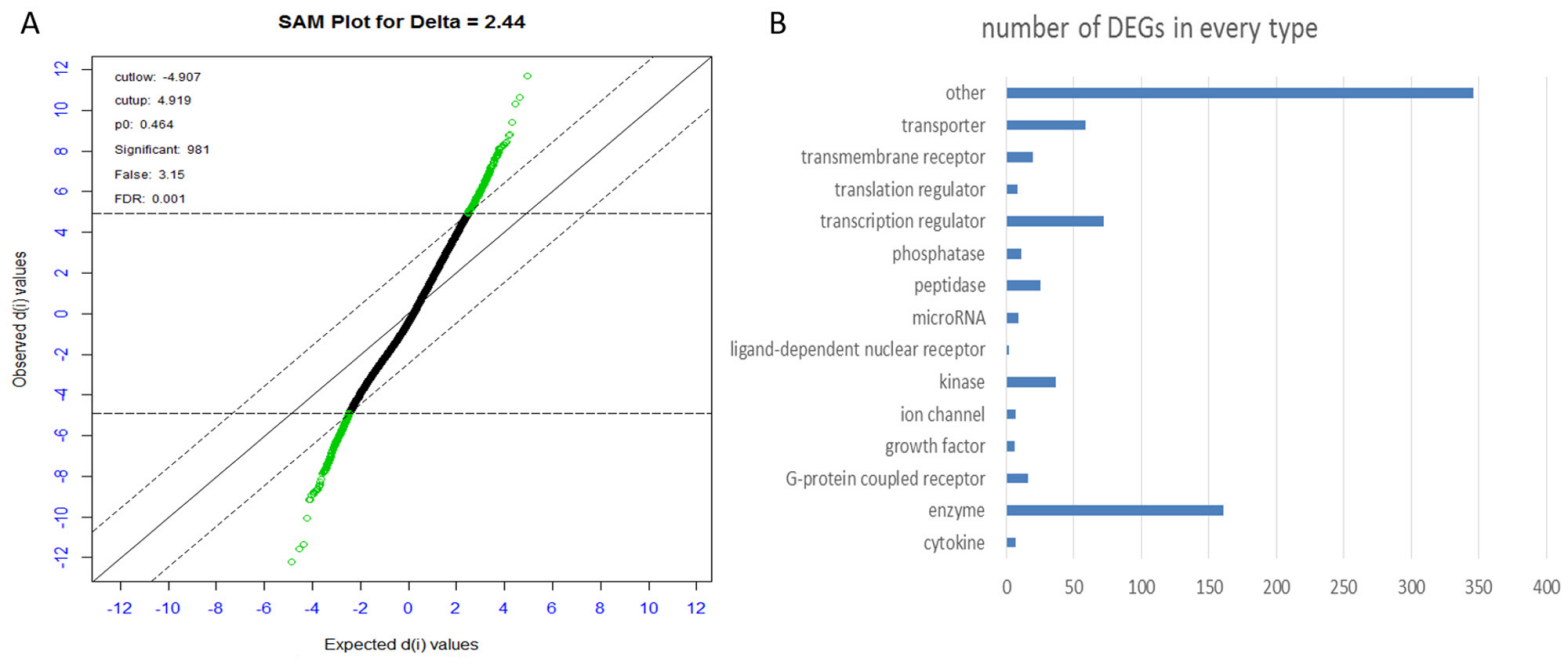
**(A)** Plot of the observed d-values vs. the ordered expected d-values. Each gene is represented by a dot, and the differentially expressed genes are colored in green. Compared to control group, there are 421 genes being significantly up-regulated (green dots above) and 376 genes being significantly down-regulated in HCC (green dots below) at an FDR of 0.1%. **(B)** Plot of the number of significant genes vs. types identified from DEGs from IPA.

A total of 981probes were screened out at the delta = 2.44 with the FDR<0.1% (Figure 2A) (Supplementary Table 1). The minimum FDR value was reserved if several probes corresponded the same gene. At last, 797 DEGs between the early HCC and normal controls were screened out using SAM, including 421 up-regulated and 376 down-regulated genes (Supplementary Table 1). All of these DEGs are classed into 14 types according to IPA as shown in Figure 2B.

According to the classification, enzymes, TFs, transporter, kinase composed most of the DEGs.

### Canonical pathway analysis

**Figure 3:**
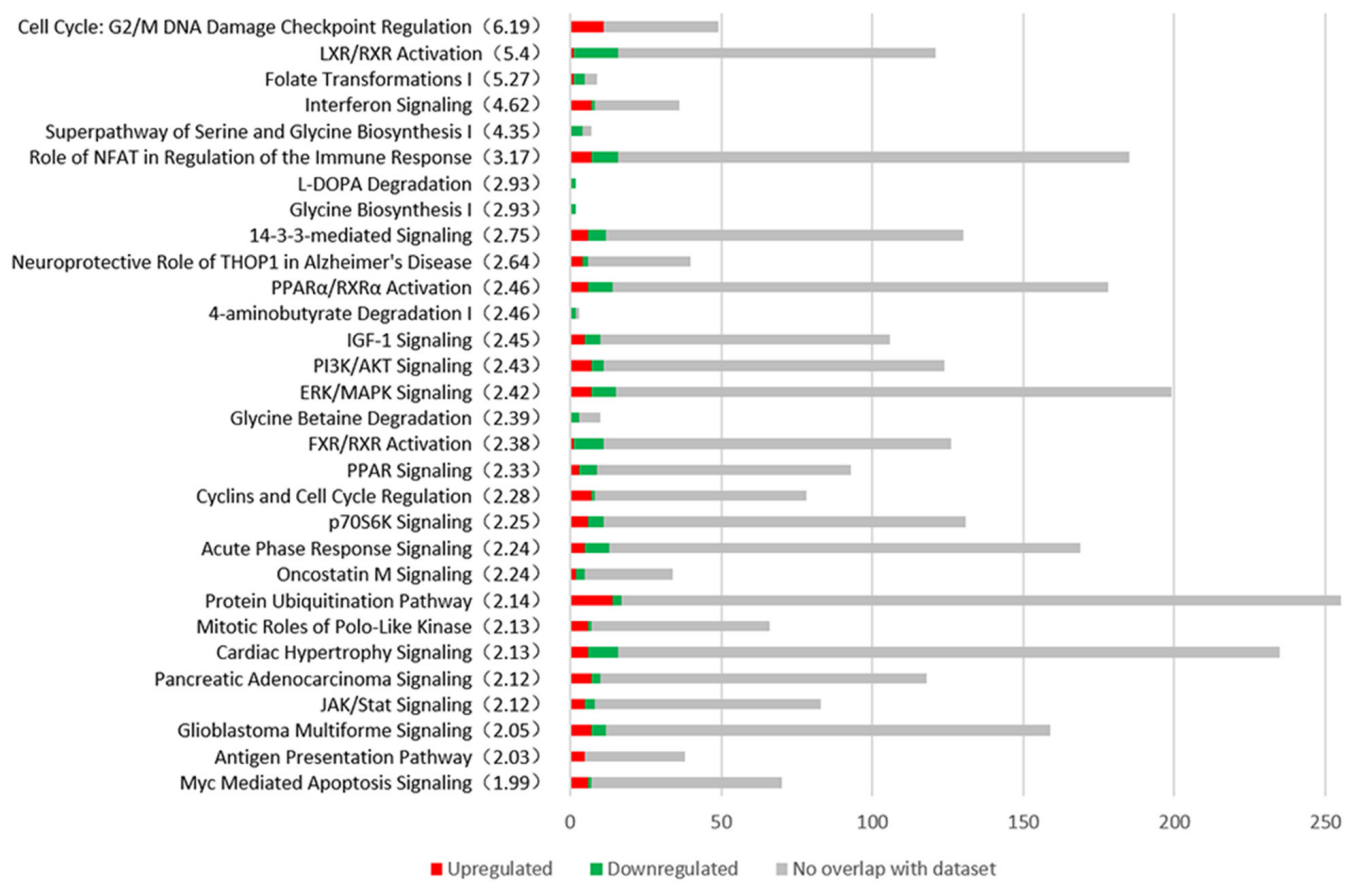
The most representative canonical pathways associated with the early stage of HCC are shown from Ingenuity Pathway Analysis (IPA) The number of DEGs are shown in the figure. Red represents the up-regulated genes, the green represents the down-regulated genes and the grey represents the no overlap genes with dataset. The significance (-log p value) of every pathway is indicated in parenthesis.

We compared the early HCC group with the control group using IPA tool. 78 canonical pathways were identified with a *p*-value<0.05 and the top 26 pathways associated with the onset of HCC are shown in Figure 3. Cell Cycle: G2/M DNA Damage Checkpoint Regulation, LXR/RXR Activation, Folate Transformations I, Interferon Signaling, Superpathway of Serine and Glycine Biosynthesis I, Role of NFAT in Regulation of the Immune Response are the most significant changed pathways in HCC. Notably, 11 genes participated in G2/M DNA damage checkpoint regulation are all up-regulated.

### The upstream regulator analysis

**Figure 4:**
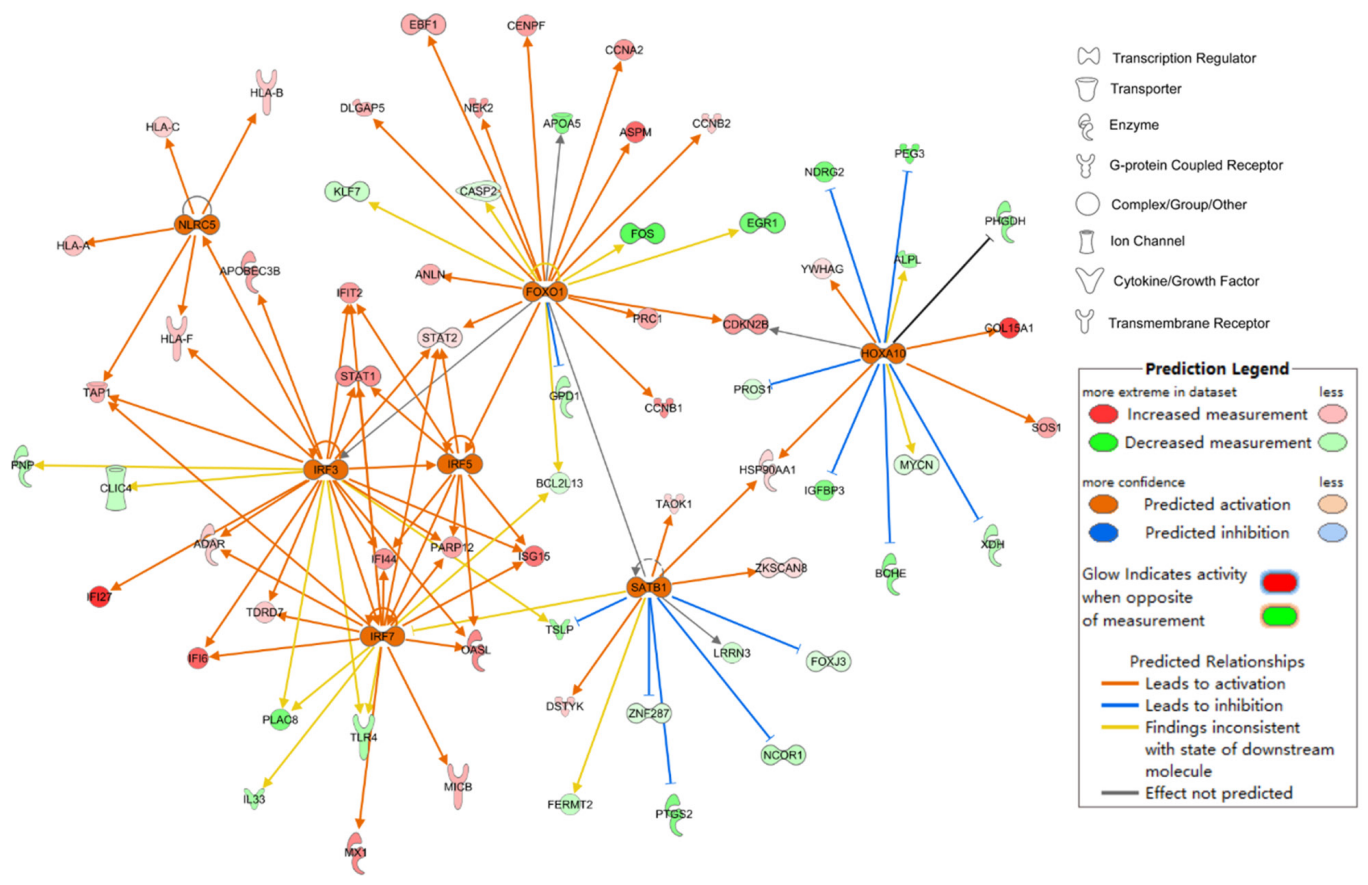
Upstream regulator analysis of differentially expressed genes at the early stage of HCC 7 TFs which was predicted to be activated as determined by IPA.

The upstream regulator analysis was performed by IPA and 7 transcription factors (TFs) were predicted to be activated and 6TFs be inhibited as shown in Table 1. The 7 predicted activated TFs and their target genes are shown in Figure 4. The DEGs regulated by FOXO1 participate in cell cycle mainly.

**Table 1:** Upstream regulator analysis of differentially expressed genes in the early stage of HCC

Upstream regulator	Predicted activation state	Activation z-score	p-Value of overlap	Target molecules in dataset
IRF3	Activated	2.248	0.0000463	ADAR,APOBEC3B,CLIC4,HLA-F,IFI27,IFI44,IFI6,IFIT2,ISG15,OASL,PARP12,PLAC8,PNP,STAT1,STAT2,TAP1,TDRD7,TLR4,TSLP
IRF7	Activated	2.367	0.0000635	ADAR,BCL2L13,IFI44,IFI6,IFIT2,IL33,ISG15,MICB,MX1,OASL,PARP12,PLAC8,STAT1,STAT2,TAP1,TDRD7,TLR4
NLRC5	Activated	2.182	0.000528	HLA-A,HLA-B,HLA-C,HLA-F,TAP1
HOXA10	Activated	2.335	0.00442	ALPL,BCHE,CDKN2B,COL15A1,HSP90AA1,IGFBP3,MYCN,NDRG2,PEG3,PHGDH,PROS1,SOS1,XDH,YWHAG
IRF5	Activated	2.607	0.0105	IFI44,IFIT2,ISG15,OASL,PARP12,STAT1,STAT2
SATB1	Activated	2.373	0.0316	DSTYK,FERMT2,FOXJ3,HSP90AA1,LRRN3,NCOR1,PTGS2,TAOK1,TSLP,ZKSCAN8,ZNF287
FOXO1	Activated	2.005	0.0357	ANLN,APOA5,ASPM,BCL2L13,CASP2,CCNA2,CCNB1,CCNB2,CDKN2B,CENPF,DLGAP5,EBF1,EGR1,FOS,GPD1,KLF7,NEK2,PRC1,STAT2
TP53	Inhibited	-2.388	1.93E-09	ABAT,ACAA2,ADGRB3,ALB,ANLN,AQP3,ASPM,ATAD2,AURKA,BMX,CAMLG,CARHSP1,CASP2,CCNA2,CCNB1,CCNB2,CD82,CDKN2A,CDKN3,CENPF,CKAP2,CLIC4,CLU,COL4A1,COMT,CXCL12,DLGAP5,DNM1L,DUT,EDIL3,EGR1,EIF4G3,ELK4,ESR1,EZH2,FAT1,FERMT2,FOS,GMNN,GNA14,H2AFY,HLA-B,HMMR,HSP90AA1,IGFBP3,ISG15,KPNA2,MAP2K1,MDM4,MELK,MX1,MYBL1,NDC80,NDRG2,NEK2,NPNT,ORM2,PDGFA,PDLIM5,PEG3,PHGDH,PIK3R3,PLPBP,PODXL,PPFIBP1,PRC1,PRKAB1,PTGS2,PTTG1,PURA,PVT1,RACGAP1,RALBP1,RFWD2,RLIM,ROBO1,RRM2,SFRP1,SON,STAT1,STEAP3,TAP1,TFPI2,TINAGL1,TJP1,TOP2A,TP53BP2,TPD52L1,TRIO,USP14,WNT2,XPO1,ZEB2
HNF1A	Inhibited	-2.256	0.0000157	ABCC9,ADH6,ALB,ANKS4B,APOH,AQP3,C8A,C8B,CYP1A2,F11,FOXJ3,HPX,IFNAR1,LCAT,LEF1,LY6E,MT1H,MT1X,NBR1,NPC1L1,NR1H4,PAMR1,PKHD1,PNO1,PPP1R1A,PZP,SLC12A7,SLC17A2,SLC38A4,SLC7A2,SUPV3L1,TMEM27,TROVE2,ZNF502
HMGA1	Inhibited	-2.206	0.000605	ALPL,COL4A1,EGR1,ESR1,FOS,GHR,IDI1,IER2,IGFALS,IGFBP3,LY6E,MAPT,PTGS2,PTH1R
TRIM24	Inhibited	-2.525	0.00217	IFI44,IFIT2,ISG15,OASL,PARP12,PLAC8,SAMHD1,STAT1,STAT2,TAP1
IRF4	Inhibited	-2.975	0.0322	ALPL,CCNB1,CDKN2A,ENTPD1,IL33,ISG15,PDCD6,SMARCA4,STAT1,STAT2
ELK1	Inhibited	-2.146	0.0329	CDKN2A,EGR1,FOS,PTGS2,TPD52L1

### Gene set enrichment analysis confirmed the enrichment of G2/M checkpoint at the early stage of HCC

**Figure 5:**
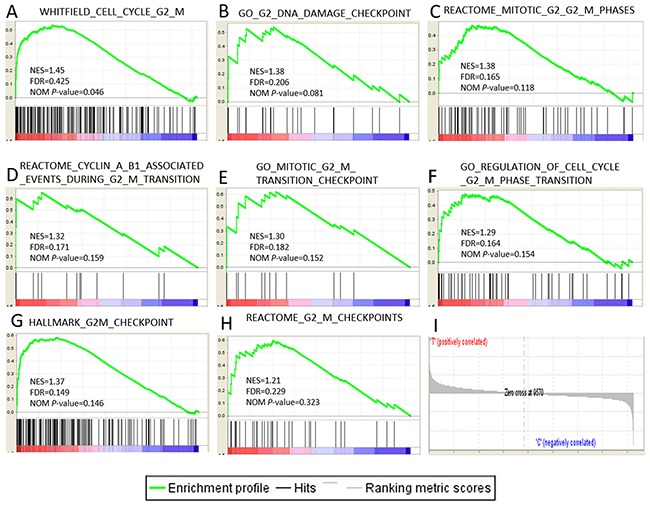
Gene expression profiling identifies pathways upregulated at the early stage of HCC **(A-H)** The 7 significantly enriched gene sets in HCC. The normalized enrichment score, the false discovery rates (FDR) and the nominal p-value score(NES) are indicated for each gene set. Each bar at the bottom of each panel represents a member gene of the respective pathway from plot A-H and **(I)** shows its relative location in the ranked list of genes.

The results from IPA showed that G2/M checkpoint regulation was the most significantly changed biological process which relates to cell proliferation closely. So we selected 14 gene sets related to the G2/M checkpoint from all 15142 gene sets in GSEA to confirm the enrichment of G2/M checkpoint or related process (Supplementary Table 2). We set the number of permutations was 1000, the permutation type was gene-set, the max and min size of gene sets selected was 500 and 10 respectively with the other parameters were default. As a result, 13/14 genes sets were up-regulated in HCC with 7 gene sets were significantly enriched at FDR<25% and one gene set was enriched at nominal p-value<0.05. 1/14 gene sets was up-regulated in the control group (BHATI_G2M_ARREST_BY_2METHOXYESTRADIOL_DN). The enrichment plot of 8 up-regulated gene sets are shown in Figure 5.

### Leading edge analysis

**Figure 6:**
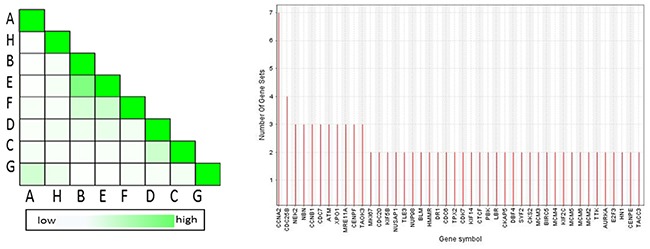
Set-to-set and gene in subsets from the leading edge analysis The left graph showed the overlap between 8 subsets: the darker the color, the greater the overlap between the subsets. The intensity of the cell A and B corresponds to an X/Y ratio which is the number of leading edge genes from set A and Y is the union of leading edge genes in sets A and B. The right graph shows each gene and the number of subsets in which it appears.

In order to determine which genes appeared frequently in 8 genes sets associated with G2/M checkpoint and explore the genes that have the highest impact on G2/M checkpoint, 8 gene sets were dedicated to perform the leading edge analysis as shown in Figure 6. Three terms from GO overlapped mostly. CCNA2 appeared in 7 gene sets, CDC25B appeared in 6 gene sets, and NEK2,NBN,CCNB1,CDC7,ATM,XPO1,MRE11A,CENPF,TAOK3 appeared in 3 gene sets.

### PPI network construction and analysis from all DEGs

**Figure 7:**
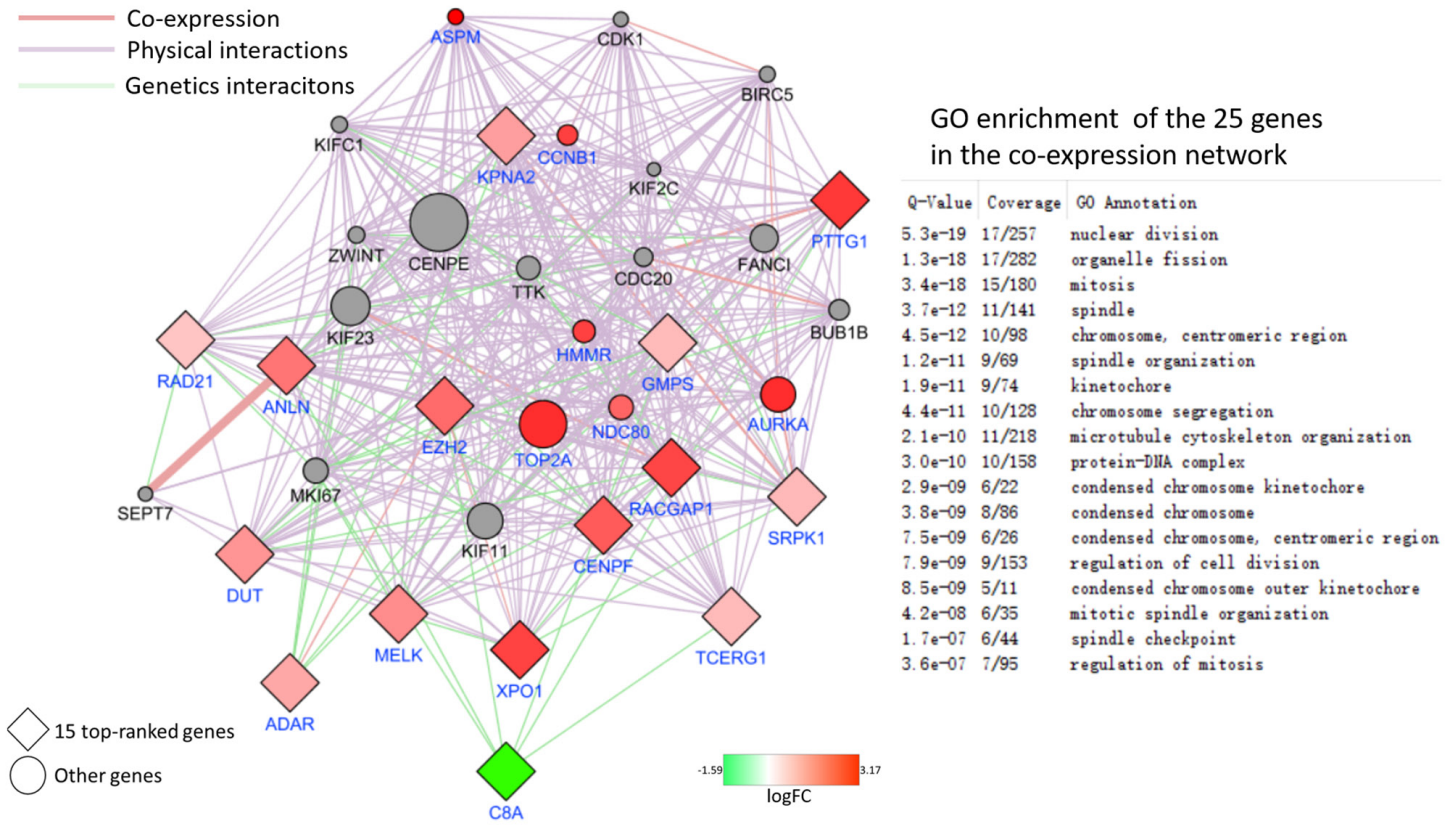
PPI network of 15 top-ranked DEGs and top 20 most related genes associated with the onset of HCC The genes belong to DEGs colored by their logFC. The network was generated using the GeneMANIA plugin. The networks legend indicates the types of interactions between genes.

From the 797 DEGs, a network with 721 nodes and 30900 edges was constructed using GeneMANIA plugin. And eleven scoring methods including the newly developed algorithms MCC were performed by use of cytoHubba plugin. At last, 15 genes were screened out according to local-based method MCC and global-based method bottleneck and stress. The co-expression network from the 15 top-ranked genes was constructed as shown in Figure 7. 14 out 15 genes were up regulated and only C8A down regulated. 6 out of 20 top related genes were DEGs and were all up regulated. Most of these genes related to cell cycle.

### Kaplan-Meier survival analysis

**Figure 8:**
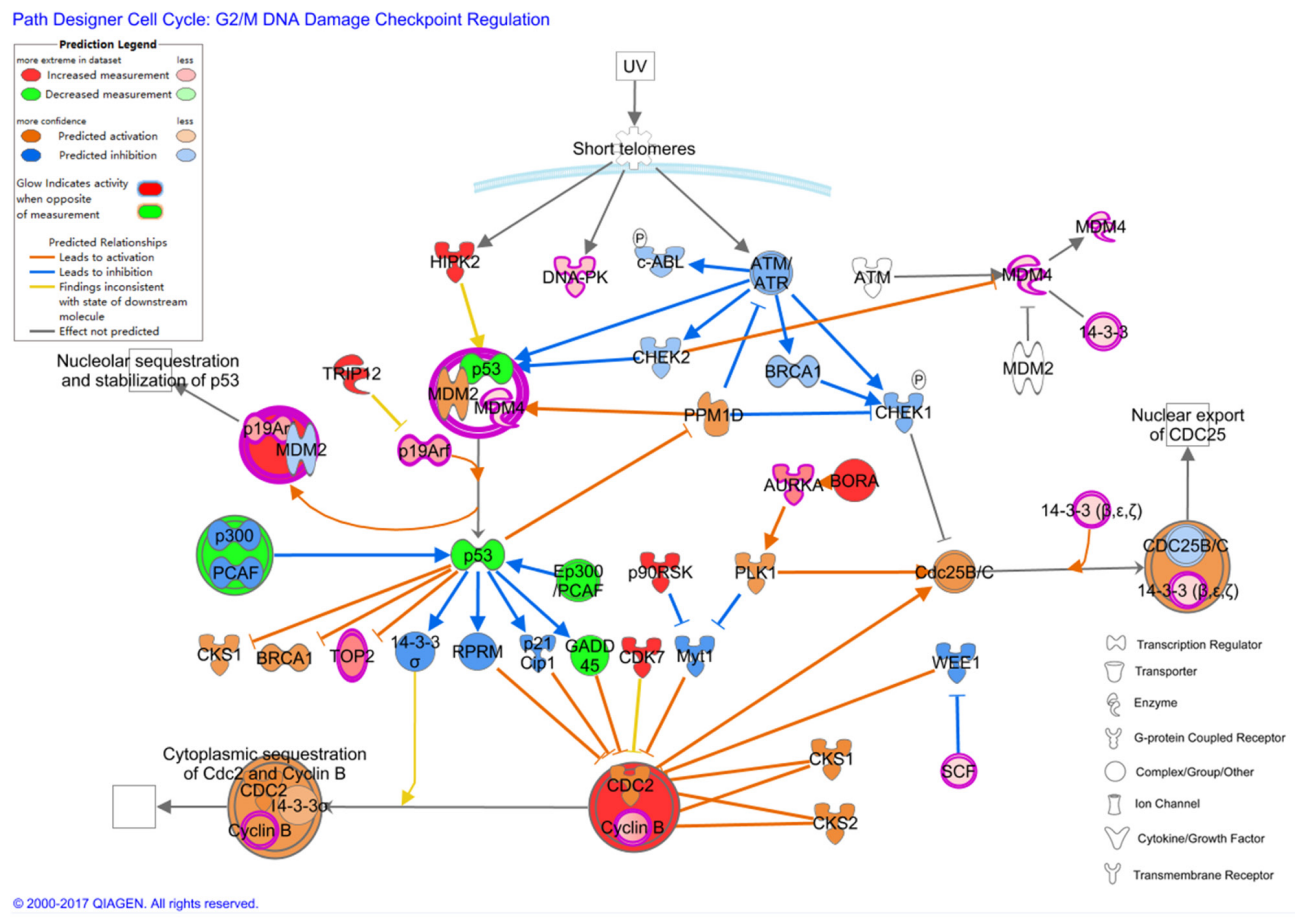
The molecular activation prediction (MAP) figure based on IPA

In order to find the relationship between the key genes and survival of the HCC patients, we performed the Kaplan-Meier Survival analysis. The data showed the High expression of XPO1, KPNA2, GMPS, MELK were correlated with high risk, poor prognosis and shorter overall survival time significantly as shown in Figure 8. Kaplan-Meier survival curves indicated the patients in high risk group had obviously shorter OS time than those in low risk(p<0.05).

## DISCUSSION

In order to explore the most significantly dysregulated pathways and key genes which play roles at the early stage of HCC and gain an insight into the onset of HCC which could be applied to the early diagnosis and therapy, a series of bioinformatics methods were performed. According to our studies, the Cell Cycle: G2/M checkpoint regulation was the most dysregulated pathways with 11 DEGs are all up regulated. As the second checkpoint within the cell cycle, G2/M checkpoint prevents cells with damaged DNA from entering the M phase so that these DNAs could be repaired. This kind of regulation is critical to prevent cells from going through malignant transformation.

The deficiency of p53 in most human cancers make G1 checkpoint defective. The S-phase checkpoint slows rather than arrest of the cell cycle. So the cancer cell with damaged DNA could accelerate through the cell cycle and arrest at the G2 checkpoint. All the above makes the G2 checkpoint an attractive therapeutic target for anticancer therapy [10]. It has been demonstrated that Polo-like kinase (PLK) may be an early diagnostic marker for the development of HCC by regulating the G2/M checkpoint [11]. lncRNA16 is a promising biomarker for early diagnosis of lung cancer by promoting the G2/M transition by regulating the transcription of cyclin B2. As a promising antitumor agent, Isocorydine(ICD) could induce G2/M cycle arrest of HCC through activation of GADD45A-p21 pathway [12]. So, we inferred the dysregulation of this pathway is very important to the onset of HCC.

**Figure 9:**
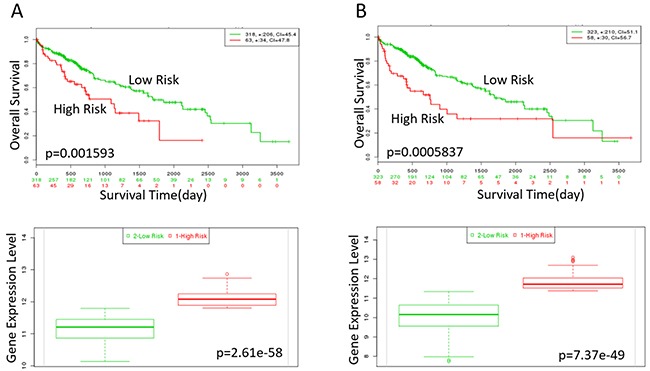
Kaplan-Meier curves of XPO1 and KPNA2 in TCGA liver cancer dataset (https://tcga-data.nci.nih.gov/publications/tcga) with SurvExpress (*n*=381) Censoring samples are shown as “+” marks.Horizontal axis represents time (day) to event. Outcome event, time scale, condordance index (CI) and p-value of the log-rank test are shown. Red and green curves represent High and Low-risk groups. The number below horizontal axis represents the number of individuals not presenting the event of the corresponding risk groups along time. **(A)** High expression of XPO1 is correlated with high risk, poor prognosis and shorter overall survival time. **(B)** High expression of KPNA2 indicates high risk, poor prognosis and shorter overall survival time. The down panel shows box plot across risk groups with the p-value.

In order to confirm the result, GSEA was performed from the gene set level. GSEA is a computational method which determines whether an a priori defined set of genes shows statically significant between two biological states at the level of gene sets instead of an individual gene. GSEA can make up for the deficiency of traditional strategies which focused on the DEGs. The result of 13 gene sets associated with G2/M checkpoint upregulated in HCC and 1 upregulated in control confirmed that the G2/M checkpoint changed significantly. In order to determine the genes which contributed most to the enrichment result of 13 gene sets, the leading edge analysis was performed. 11 genes including CCNA2 (DEG), CDC25B, NEK2 (DEG), NBN, CCNB1(DEG), CDC7, ATM, XPO1(DEG), MRE11A, CENPF(DEG), TAOK3 appeared most often in several gene sets. The transcriptional factor FOXO1 was predicted active and the target genes regulated by it were associated with cell cycle most. The crosstalk of genes participated in the G2/M checkpoint is shown in Figure 9. from the molecular activity predictor(MAP).

As a plugin in cytoscape, cytoHubba provides an effective method to identify important nodes in biological networks. It could accomplish the computation of eleven methods in one stop shopping way including four local-based methods and seven global-based methods with the Maximal Clique Centrality (MCC) is a new method in order to increase the sensitivity and specificity. Through the combination of MCC, bottleneck and stress, 15 DEGs are screened out including SRPK1, XPO1, GMPS, MELK, DUT, TCERG1, RAD21, CENPF, PTTG1, EZH2, ANLN, KPNA2, RACGAP1, ADAR, C8A. All the above genes except C8A are upregulated and most of them participate in the cell cycle. From the analysis of co-expression network, we can find that the co-expressed genes which belong to DEGs upreguated too. Notably, XPO1 and CENPF is also screened out in the leading edge analysis.

SR (serine/arginine-rich domain) proteins play a critical role in many process including nuclear export of mature mRNA, polymerase II transcription and nonsense-mediated mRNA decay. SRPK1 (Serine/threonine-protein kinase) can phosphorylate SR proteins through its PKc superfamily kinase domain. But, SRPK1 displays pleiotropic effects in various cancers and regulates different cellular properties which might be related to preferential activation of different downstream signaling pathways [13–20]. Expression of SRPK1 was significantly upregulated frequently in HCC cell lines and HCC samples compared with the normal tissue sample both at the mRNA and protein level [21]. In the study of HCC, SRPK1 may be located downstream of AKT and activated AKT may induce the autophosphorylation of SRPK1 which lead to the phosphorylation of downstream splicing factors [20]. SRPK1 may be associated with FAK signaling, MAPK signaling, Wnt/β-catenin signaling and angiogenesis [13, 22]. More research implied that SRPK1 may be a novel target for cancer diagnosis and therapy. But the detailed roles and mechanisms of SRPK1 in cancer especially in HCC are not clear.

KPNA2 (karyopherin alpha 2) may participate in carcinogenesis by regulating the translocation of some cargo proteins which are involved in cancer. It was demonstrated that KPNA2 promotes cell proliferation and tumorigenicity in epithelial ovarian carcinoma by regulating c-Myc and FOXO3a [23]. The knockdown of KPNA2 could inhibit proliferation of several cancer cells including liver and lung and KPNA2 may be a useful prognostic biomarker to monitor cancer prognosis [24, 25].

To date, over 230 kinds of proteins were verified as the cargo of XPO1(Chromosome region maintenance 1,CRM1) including p53, p21, IkB, ribosomal subunits and so on [26]. XPO1 plays a vital role in nucleo-cytoplasmic transport through RanGTP dependent mechanism. For most typical tumor suppressive proteins such as p21, they play different functions according to their subcellular localization which elucidate that XPO1 plays an vital role in the process of cancer and may lead to a new method for cancer therapy which associated with cell cycle arrest and induction of apoptosis [27–29].

As glutamine amidotransferases involved in de novo purine biosynthesis, GMPS (GMP synthetase) was shown to have a striking role in cell proliferation. Under genomic stress, GMPS plays a vital role in the relay of p53 stabilization by TRIM21-GMPS-USP7 molecular cascade. The guanine nucleotides is essential for nucleotide formation, energy storage and nuclear transport which could be provided by guanine biosynthesis pathway in cancer cells [30, 31]. Repression of GMPS by p53 through p21 is a functionally relevant part of the p53-mediated process in inhibiting tumor cell growth in liver cancer [32].

It is supposed that MELK (maternal embryonic leucine zipper kinase) plays critical roles in many aspects including cell cycle, cell proliferation, embryogenesis and oncogenesis due to its overexpression in many kinds of cancers. MELK is associated with early HCC recurrence and poor patients’ survival but the mechanism has not been elucidated [33]. MELK knockdown or deletion in GC (gastric cancer) and ovarian cancer cells activates G2/M arrest and enhances apoptosis [34, 35].

Previous studies have revealed that amplified CENPF (centromere protein F) may play a role as common cancer-driver genes in human cancers. CENPF contains many leucine zipper motifs and is regulated in a cell cycle-dependent manner [36]. It amplified and overexpressed not only in HCC but also in many other types of human cancer including breast cancer, colorectal cancer, prostate cancer [37, 38]. Silence of CENPF arrests HCC cells at the G2/M transition with the accumulation of MPF (mature promoting factor) and CCNB1/CDC2 complex [39]. In consistence with these studies, the present study found that CENPF is identified as a key gene not only in DEGs but also in leading edge analysis which based on gene set level.

As the single down regulated one in all the key genes, C8A (complement component alpha) involves in the complement system and participates in the formation of MAC (membrane attack complex) combined with other complement proteins such as C5b, C6, C7, C8 and C9. In addition, the expression of several other complement components or subunits are all down regulated ,including C1S, C2, C5, C6, C7, C8B, C8G, C9. Obviously, it is not coincidence. That is to say, the activity of complement system down regulated at the early stage of HCC. The transcription of C8A and C5 is regulated by HNF1α (hepatocyte nuclear factor 1 alpha) both are essential components of MAC [40]. The relationship between C8A and cancer is not clear yet.

G2/M checkpoint provides an opportunity for DNA repair by increasing the time for repair and by transcriptionally inducing gene expression and stopping the proliferation of damaged cells [41]. To the best of our knowledge, G2/M checkpoint transition activated at the early stage of HCC was provided for the first time through the microarray analysis. The negligent G2/M checkpoint enhanced the possibility for DSB (double strand breakage). And the unrepaired DSBs before mitosis will pose a higher risk for genomic instability and tumor cell development [42]. So, the defect of G2/M checkpoint may play a critical role at the onset of HCC.

## MATERIALS AND METHODS

### Data source

The gene expression dataset GSE6764 were downloaded from the Gene Expression Omnibus (GEO) database by Wurmbach E et.al (https://www.ncbi.nlm.nih.gov/geo/). 75 tissue samples obtained from patients undergoing resection or liver transplantation were divided into 8 groups from pre-neoplastic lesions to HCC and normal liver were used as control (http://www.ncbi.nlm.nih.gov/geo/). We combined very early and early HCC to the case group including 18 tissue samples altogether, and the control group, with 10 normal liver tissue. All tissue samples are hybridized on the human U133 plus 2.0 array (Affymetrix).

### Microarray data analysis and identification of differentially expressed genes (DEGs) using SAM

Robust Multi-array Average (RMA) algorithm including background correction, normalization and summarization was performed to convert the .CEL raw file to expression data which based on R language [43]. The simpleaffy package was utilized to perform the quality control. Once the signal value for each probe set calculated in every microarray, the t-test based significance analysis of microarrays (SAM) which make use of permutations to simulate for every gene a situation in which there is no difference between the two groups was utilized to determine the DEGs. SAM method adjusts the p-value to false discovery rate (FDR) to reduce the false-positive through multiple testing. A <0.1% False discovery rate (FDR) cut-off was used for all differential expression calculations [44].

### Ingenuity pathway analysis

Ingenuity Pathway Analysis (IPA) is a functional analysis tool (Ingenuity Systems, Mountain view, CA, USA). We use IPA to identify the most significant pathways (including 302 metabolic pathways and 360 signaling pathways) and construct molecular interaction networks from the DEGs. In brief, we uploaded the DEGs list file containing gene symbols, FC, *p*-values to IPA and performed the core analysis. In general settings, the node types, data sources, confidence, species, tissues & cell lines and mutation were specified.

### The IPA upstream transcriptional regulator analysis

In order to explain the biological activities due to the DEGs, we identified the cascade of upstream transcriptional regulators with p-value of overlap <0.05 and the absolute activation z-score>2.

### Gene set enrichment analysis (GSEA) and leading edge analysis

GSEA is a kind of gene enrichment method considering the full list of genes different from single gene method [45]. In GSEA, genes are ranked by their correlation with phenotype and every enrichment gene set will get an enrichment score (ES). In this study, 2000 gene permutations were used to generate a null distribution for ES, then each pathway will attain a normalization enrichment score(NES). Gene sets with considered significantly enriched with a relatively relax *p*-value and FDR<0.25. A leading edge analysis was performed to elucidate key genes associated with the early stage of HCC, especially the G2/M checkpoint regulation [46].

### Construction of PPI network from all DEGs and the screening of key genes

In order to comprehend the specific molecular mechanism of early HCC, we constructed the PPI network based on GeneMANIA plugin and calculated the key DEGs using cytoHubba plugin [47, 48]. At last, we built the co-expression network of top-ranked genes from all DEGs and performed the visualization and analysis by use of Cytoscape 3.4.0(http://cytoscape.org/).

### Kaplan-Meier survival analysis

SurvExpress(http://bioinformatica.mty.itesm.mx:8080/Biomatec/SurvivaX.jsp) was employed to perform the survival analysis in the datasets TCGA-liver cancer containing 422 samples provided by SruvExpress using the key genes as an input. For the duplicated genes, all probe sets/records will be averaged per sample using the original (Quantile-Normalized) data. The maximum risk groups were selected for the cox survival analysis. This method uses an optimization algorithm from the ordered PI to produce risk groups as described in the tutorial provided in SurvExpress website [49].

## CONCLUSION

The combinatorial effect of the GSEA, DEGs, and leading edge analysis output shed a light on the elucidating of key pathways and genes which genetically dysregulated at the early stage of HCC. The study unveiled that the G2/M checkpoint plays a vital role at the onset of HCC. And the genes SRPK1, XPO1, GMPS, MELK, DUT, TCERG1, RAD21, CENPF, PTTG1, EZH2, ANLN, KPNA2, RACGAP1, ADAR, C8A could be considered as critical genes for this process. These findings contributed to a better understanding of the onset of HCC. Further studies were required to elucidate the mechanism of the process.

## SUPPLEMENTARY MATERIALS TABLES






