# Supplementary material for: G2/M checkpoint plays a vital role at the early stage of HCC by analysis of key pathways and genes
Source: Oncotarget. 2017 Jul 18;8(44):76305–17. doi: 10.18632/oncotarget.19351 (PMC5652707; doi:10.18632/oncotarget.19351)
Supplement: Supplementary file 1 [file oncotarget-08-76305-s001.pdf]

G2/M checkpoint plays a vital role at the early stage of HCC by analysis of key pathways and genes

SUPPLEMENTARY MATERIALS

Supplementary Table 1: The details of DEGs 18

See Supplementary File 1

Supplementary Table 2: Gene set related to the G2/M checkpoint 17

See Supplementary File 2
